# Deletion of *Irf4* in T Cells Suppressed Autoimmune Uveitis and Dysregulated Transcriptional Programs Linked to CD4^+^ T Cell Differentiation and Metabolism

**DOI:** 10.3390/ijms22052775

**Published:** 2021-03-09

**Authors:** Minkyung Kang, Hyun-Su Lee, Jin Kyeong Choi, Cheng-Rong Yu, Charles E. Egwuagu

**Affiliations:** 1 Molecular Immunology Section, Laboratory of Immunology, National Eye Institute (NEI), National Institute of Health, Bethesda, MD 20892, USA; minkyung.kang@nih.gov (M.K.); hyunsu.lee@kmu.ac.kr (H.-S.L.); appieim@daum.net (J.K.C.); YuC@nei.nih.gov (C.-R.Y.); 2 Department of Immunology, Jeonbuk National University Medical School, Jeonju, Jeonbuk 54907, Korea

**Keywords:** CD4, IRF4, Autoimmune Uveitis, IkZFs, IL35, metabolism

## Abstract

Interferon regulatory factor-4 (IRF4) and IRF8 regulate differentiation, growth and functions of lymphoid and myeloid cells. Targeted deletion of *irf8* in T cells (CD4-IRF8KO) has been shown to exacerbate colitis and experimental autoimmune uveitis (EAU), a mouse model of human uveitis. We therefore generated mice lacking *irf4* in T cells (CD4-IRF4KO) and investigated whether expression of IRF4 by T cells is also required for regulating T cells that suppress autoimmune diseases. Surprisingly, we found that CD4-IRF4KO mice are resistant to EAU. Suppression of EAU derived in part from inhibiting pathogenic responses of Th17 cells while inducing expansion of regulatory lymphocytes that secrete IL-10 and/or IL-35 in the eye and peripheral lymphoid tissues. Furthermore, CD4-IRF4KO T cells exhibit alterations in cell metabolism and are defective in the expression of two Ikaros zinc-finger (IKZF) transcription factors (Ikaros, Aiolos) that are required for lymphocyte differentiation, metabolism and cell-fate decisions. Thus, synergistic effects of IRF4 and IkZFs might induce metabolic reprogramming of differentiating lymphocytes and thereby dynamically regulate relative abundance of T and B lymphocyte subsets that mediate immunopathogenic mechanisms during uveitis. Moreover, the diametrically opposite effects of IRF4 and IRF8 during EAU suggests that intrinsic function of IRF4 in T cells might be activating proinflammatory responses while IRF8 promotes expansion of immune-suppressive mechanisms.

## 1. Introduction

The interferon regulatory factor (IRF) family of transcription factors is comprised of nine members that play critical roles in regulating cell growth and development [1,2]. The family is characterized by an N-terminal DNA-binding domain (DBD) that recognizes the core sequence (5-GAAA-3) of the canonical IFN-stimulated response element (ISRE). Although IRFs are ubiquitously expressed, IRF4 and IRF8 are abundant in lymphocytes and play important role in host immunity by regulating the differentiation and growth of lymphoid and myeloid cells [3]. IRF4 and IRF8 are closely related and functionally distinct from other IRFs by the relatively low binding affinity of their DBD to the core 5′-GAAA-3′ of the ISRE motif [4]. Both factors regulate gene transcription by interacting with POU.1 or AP-1 factors FOS/JUN and the basic leucine zipper transcription factor ATF-like (BATF) factors. These factors recruit IRF4 or IRF8 to ETS-IRF composite motifs (EICE) or AP-1/BATF-IRF composite elements (AICEs) of target genes [5,6,7,8,9]. The role of IRF4 or IRF8 in lymphocyte development and effector functions suggest that they are potential therapeutic targets for modulating immune responses during autoimmune diseases.

The Ikaros Zinc Finger Transcription Factors (IkZF) regulate T cell differentiation and they induce expression of IRF4 during early stages of lymphoid cell development and lymphopoiesis [10,11,12]. The IKZF family is comprised of Ikaros, Helios, Aiolos and Eos, and depending on stage of lymphoid cell development, they can function as transcriptional repressors or activators [13]. In B cells, the IkZF transcription factors, Ikaros and Aiolos are IRF4 binding partners and binding of Ikaros/IRF4 complexes to zinc finger-IRF composite elements (ZICEs) during plasma cell differentiation mediate repression of a subset of genes that antagonize plasma cell development [14]. In zebra fish, IRF4 is also a direct target of Ikaros and genetic ablation of either *irf4* or *ikaros1* abrogates embryonic T-lymphopoiesis [15]. In vertebrates, targeted deletion of DNA binding or dimerization domain of IkZFs family members results in lack of the lymphoid lineage [13]. On the other hand, IRF4 plays essential role in T-helper cell differentiation, TCR affinity-mediated metabolic programming and T cell clonal expansion [16,17,18]. IRF4 also interacts with lymphocyte lineage-specifying transcription factors including STAT3, FOXP3, RORγt and regulates the differentiation of T-helper subsets [16,19]. Taken together, these observations underscore the critical roles IkZF and IRF4 transcription factors in the genetic networks of lymphocytes and suggest that IkZF-induced expression of IRF4 in T cells may serve as developmental checkpoint linking metabolism to the regulation of T-helper cell differentiation and developmental programs.

Mice with global deletion of *irf4* develop progressive, generalized lymphadenopathy by 4 to 5 weeks of age, establishing the requirement of IRF4 for the function and homeostasis of mature B and T lymphocytes [20]. Although studies of *Irf4* knockout mice indicate skewing of T-helper subsets from Th1 and Th17 towards Th2, few studies have examined intrinsic and extrinsic functions of IRF4 produced by T cells in T cell-mediated autoimmune diseases such as uveitis [16]. In this study, we generated mice with targeted deletion of *irf4* in the CD4 T cell compartment and show that loss of IRF4 in CD4^+^ T cells conferred resistance to uveitis. Disease protection derived from dysregulation of lymphocyte development, alteration of T lymphocyte metabolic program and expansion of IL-10 and IL-35 expressing lymphocytes.

## 2. Results

### 2.1. Generation and Characterization of Mice with Targeted Deletion of irf4 in T Cells

**Figure 1:**
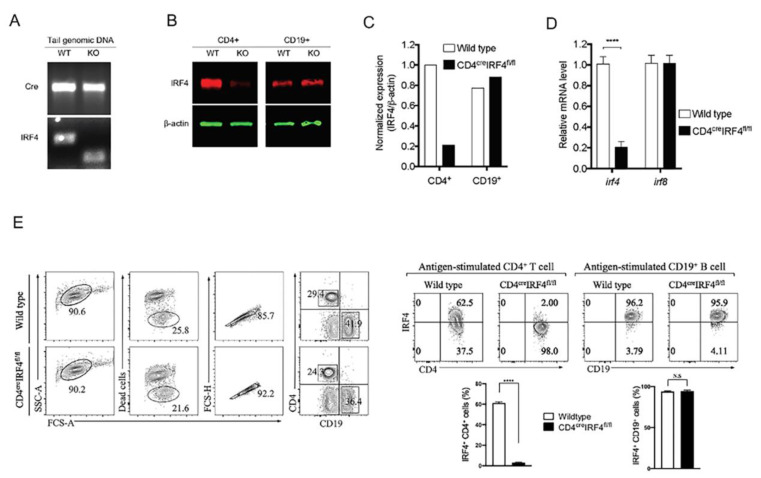
Generation and characterization of mice with targeted deletion of *irf4* in T cell. (**A**) *Irf4*^fl/fl^ mice were crossed with CD4-Cre mice to generate mice deficient of IRF4 in CD4^+^ T cells (CD4-IRF4KO). The homozygote KO mice (CD4^cre^IRF4^fl/fl^) were identified by PCR analysis of mouse tail genomic DNA. (**B**,**C**) Purified CD4^+^ T cells or CD19^+^ B cells from lymph nodes or spleen of wild-type (WT) or CD4-IRFK4O mice were analyzed for IRF4 expression by Western blot (**B**) or RT-PCR (**C**) analysis. Images of the full-length Western blot gels are provided (Supplementary Figure S1). (**D**) CD4^+^ T cells from LN and spleen of WT or CD4-IRF4KO mice were stimulated with anti-CD3/anti-CD28 for 3 days and analyzed by RT-PCR. (**E**) CD4^+^ T cells from LN/spleen and splenic CD19^+^ B cells of WT or CD4-IRF4KO mice were isolated 21 days after immunization with IRBP/CFA and analyzed by intracellular cytokine staining assay. Results represent more than 3 independent studies **** *p* < 0.0001. N.S.: Not significant

To investigate whether expression of IRF4 by T cells contributes to mechanisms of T cell differentiation and immune regulation during an autoimmune disease, we generated mice with targeted deletion of *irf4* in CD4^+^ T cells (CD4-IRF4KO). PCR analysis of tail DNA of mice derived from mating CD4-Cre and *irf4*^fl/fl^ mouse strains (C57BL/6J background) identified the CD4-Cre/IRF4^fl/fl^ (CD4-IRF4KO) mouse strain lacking *irf4* in T cells (Figure 1A). The strain was established and maintained by several cycles of “brother-sister” mating, and for all experiments described here, the phenotype was confirmed by Western blot analysis showing that the CD4-IRF4KO T cells did not express IRF4 (Figure 1B, left panel). We ascertained that the loss of IRF4 is restricted to T cells and does not extend to other immune cell types by Western blot analysis showing that IRF4 expressing is not affected in CD19^+^ B cells of the CD4-IRF4KO mice (Figure 1B, right panel). RNA analysis confirmed that loss of IRF4 is restricted to CD4^+^ T cells (Figure 1C) and that loss of *irf4* did not affect expression of IRF8 by B cells, underscoring specificity of the Cre-Lox mediated deletion process (Figure 1D). IRF4 is constitutively expressed at very low levels in resting T cells but are triggered to significantly upregulate IRF4 expression in response to TCR activation [3]. Consistent with the loss of IRF4 expression in CD4-IRF4KO T cells, intracellular cytokine staining analysis shows that more than 62.5% of activated WT T cells express IRF4 while the CD4-IRF4KO T cells exhibit a significant defect in IRF4 expression (Figure 1E, left panel). On the other hand, activated CD19^+^ B cells from the spleen of WT and CD4-IRF4KO mice expressed equivalent levels of IRF4 (Figure 1E, right panel). 

### 2.2. Deletion of irf4 in T Cells Confers Protection against Experimental Autoimmune Uveitis (EAU)

**Figure 2:**
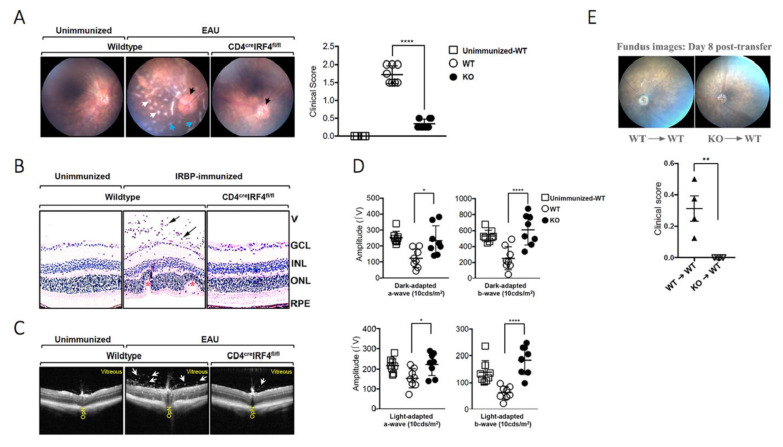
Inducible deletion of IRF4 in CD4^+^ T cells confers protection from experimental autoimmune uveitis (EAU). EAU was induced by immunization of C57BL/6J or CD4-IRF4KO mice with the uveitogenic peptide, IRBP^651−670^ in complete Freund’s adjuvant (CFA) (n = 12). Disease progression was monitored and assessed by fundoscopy, histology, optical coherence tomography (OCT) and electroretinography (ERG). (**A**) Fundus images of the retina at day 21 after EAU induction were taken using an otoendoscopic imaging system. Black arrow indicates inflammation with blurred optic disc margins and enlarge juxtapapillary areas; blue arrows indicate retinal vasculitis; white arrows indicate yellow-whitish retinal and choroidal infiltrates. Severe uveitis was observed in the WT compared to the CD4-IRF4KO mouse eyes as indicated by the clinical EAU scores. (**B**) Histologic sections are of eyes harvested 21 days after immunization with IRBP peptide and H&E staining reveal substantial numbers of inflammatory cells in the vitreous of WT compared to the CD4-IRF4KO eyes. EAU in the WT mice is characterized by the development massive retinal in-folding (*), a hallmark feature of severe uveitis. V, vitreous; GCL, ganglion cell layer; INL, inner nuclear layer; ONL, outer nuclear layer; RPE retinal pigmented epithelial layer. Black arrows, lymphocytes; Asterisks, retinal folds. (**C**) Representative OCT images show marked increase of inflammatory cells (white arrows) in the vitreous and optic nerve (OPN). (**D**) ERG analysis of the retina on day 20 after EAU induction. The averages of light- or dark-adapted ERG a-wave or b-wave amplitudes are plotted as a function of flash luminance. Data presented as the mean ± SEM of four mice in each group of at least three independent experiments. (**E**) Cells from lymph nodes and spleen of IRBP_651-670_ immunized WT or CD4-IRF4KO mice were re-stimulated ex vivo and 1 × 10^7^ cells were adoptively transferred to naïve WT mice. Disease scores determined by masked investigators indicate reduced EAU symptoms in mice that received CD4-IR4KO cells. Results represent 3 independent studies. * *p* < 0.05; ** *p* < 0.01, **** *p* < 0.0001.

We induced experimental autoimmune uveitis (EAU) in WT C57BL/6J mice (control) and CD4-IRF4KO mice by immunization with the immunopathogenic uveitogenic peptide, IRBP_651-670_ in CFA emulsion and investigated whether targeted deletion of *irf4* in CD4^+^ T cells would affect the development or severity of uveitis. In the mouse EAU model, uveitis generally manifests between day-16 and day-22 post-immunization (p.i). We therefore monitored progression and severity of the disease during this time period by fundoscopy, histology, optical coherence tomography (OCT) and electroretinography (ERG). Fundoscopic images obtained on day 21 p.i. show characteristic features of uveitis which include papillitis, retinal vasculitis with moderate cuffing, enlarged juxtapapillary areas, choroiditis, yellow-whitish retinal and choroidal infiltrates (Figure 2A). In contrast, disease in the eyes of CD4-IR4KO mice was relatively mild with very low EAU clinical scores (Figure 2A). Histology of the WT retinas 21 days p.i also revealed severe EAU, characterized by infiltration of large numbers of inflammatory cells into the vitreous, destruction of retinal cells and development of retinal in-folding, a hallmark of severe uveitis (Figure 2B). OCT, a noninvasive procedure for visualizing microstructure of the retina, revealed accumulation of inflammatory cells in the vitreous and optic nerve head of the WT but not the retina of CD4-IRF4KO (Figure 2C). Electroretinogram (ERG) measures changes in electrical potentials in response to light stimulation of the retina and is a well-established tool for detecting alterations in visual function during intraocular inflammation [21,22]. The two major ERG waves are the photoreceptor-derived a-wave and the b-wave that derive from bipolar cells in the inner nuclear layer (INL). ERG under light-adaptive stimuli reflects cone-driven signaling, while dark-adapted b-wave responses represent rod-driven signaling. Analysis of light-adapted or dark-adapted ERG on day 20 postimmunization show significantly lower a- and b-wave amplitudes in the eyes of WT compared to CD4-IRF4KO mice (Figure 2D). This suggests significant visual impairment in WT mice with EAU and consistent with lower clinical pathological score in CD4-IRF4KO EAU mice (Figure 2A,B). We performed adoptive transfer experiments to further confirm that the resistance of CD4-IRF4KO mice to EAU is a direct consequence of loss of IRF4 expression by CD4^+^ T cells. Cells from lymph nodes and spleen of IRBP_651-670_ immunized WT or CD4-IRF4KO mice were re-stimulated ex vivo, and 1×10^7^ cells were adoptively transferred to naïve WT mice and EAU scores determined by masked investigators. Adoptive transfer of the activated IRBP-specific CD4^+^ T cells from WT mice induced EAU, while mice that received activated IRBP-specific CD4^+^ T cells from CD4-IRF4KO mice did not develop EAU (Figure 2E), underscoring requirement of IRF4 expression by T cells for the development of EAU in mice.

### 2.3. Deletion of irf4 Altered Pattern of Cytokine Secretion in CD4-IRF4KO Mice during EAU 

**Figure 3:**
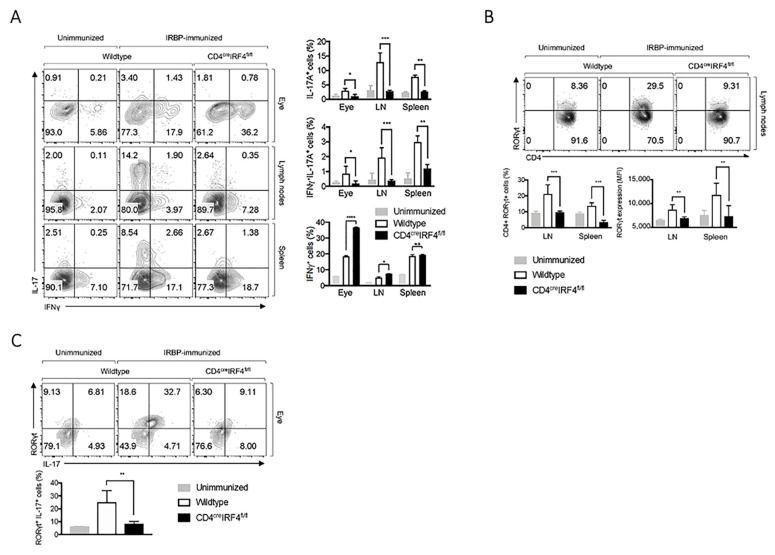
Th1 and Th17 cells are reduced in CD4-IRF4KO mice during EAU. CD4^+^ T cells from the eye, lymph nodes or spleen of WT and CD4-IRF4KO mice immunized with IRBP/CFA were sorted and analyzed by the intracellular cytokine staining assay. Numbers in quadrants represent percentage CD4^+^ T cells expressing IL-17 and/or IFN-γ (**A**), ROR-γt (**B**) or ROR-γt and IL-17 (**C**). Data represent at least three independent experiments and were analyzed using Student’s *t*-test (two-tailed). * *p* < 0.05; ** *p* < 0.01; *** *p* < 0.001. ****: *p* < 0.0001.

EAU is a CD4^+^-T cell mediated intraocular inflammatory disease mediated by aberrant recruitment of IFN-γ-producing (Th1) and/or IL-17-γ-producing Th17 cells into the retina [23]. We therefore isolated lymphocytes from the eye, LN or spleen of mice 21 days postimmunization with IRBP/CFA. Intracellular cytokine staining analysis shows significant increase of IL-17-producing CD4^+^ T cells in the eye, lymph nodes and spleen of WT compared to CD4-IRF4KO mice (Figure 3A). Moreover, we observed marked reduction of the levels of Th17 cells expressing both IL-17 and IFN-γ (DP-Th17) and the DP-Th17 population has been implicated in several organ-specific autoimmune diseases [23,24]. Further analysis of the cells by intracellular cytokine staining confirmed that the cells expressed ROR-γt, a transcription factor required for Th17 development. These results indicate that the observed IL-17-producing cells were indeed of the Th17 subset (Figure 3B,C). It is of note that the decrease of Th17 and DP-Th17 was accompanied by a concomitant increase in expansion of Th1 cells in the eye and lymph nodes of the CD4-IRF4KO mice (Figure 3A), and these observations are consistent with reports that mice with global deletion of *irf4* exhibit dysregulation in the developmental programs of several T-helper subsets [16,19]. 

### 2.4. IL-10 and/or IL-35-Producing Lymphocytes Are Expanded in CD4-IRF4KO Mice during EAU

**Figure 4:**
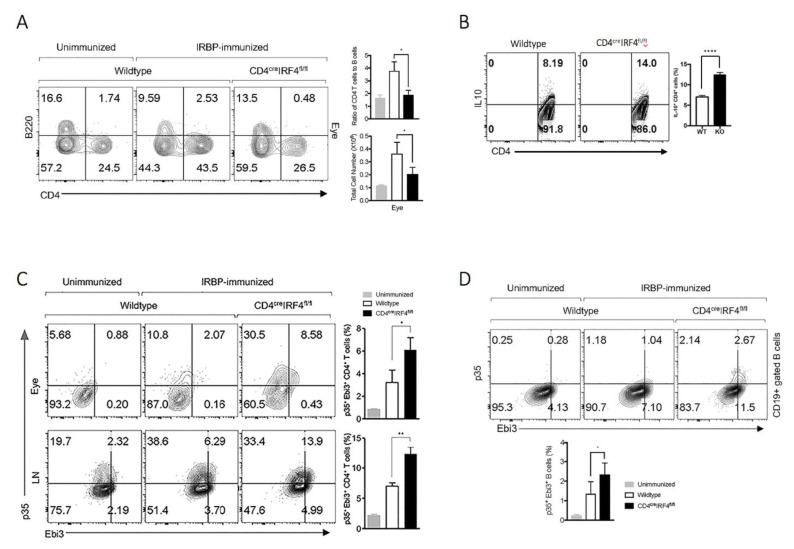
Regulatory T and B cells that produce IL-10 or IL-35 are expanded during EAU in CD4-IRF4KO mice. (**A**) Lymphocytes in the eyes of WT or CD4-IRF4KO mice immunized with IRBP/CFA were stained with CD4 or B220 Abs and numbers in quadrants represent percent B cells and T cells. (**B**–**D**) CD4^+^ T cells in eyes or lymph nodes (**B**,**C**) or B220^+^ B cells in the spleen of EAU mice (**D**) were analyzed by intracellular cytokine staining assay. Numbers in quadrants represent percent CD4^+^ T cells expressing IL-10 (**B**) or IL-35 (**C**) or B cells secreting IL-35 (**D**). Data represent three independent experiments. Analysis was performed by Student’s *t*-test (two-tailed). * *p* < 0.05; ** p < 0.01; **** p < 0.0001.

Because IL-10 and IL-35 play critical roles in regulating immunity during autoimmune diseases [25,26], we investigated whether capacity of CD4-IR4KO T cells to produce these anti-inflammatory cytokines might also be altered during EAU. Consistent with phenotype of the CD4-IRF4KO mouse, percentage of CD4^+^ T cells was reduced with no discernible difference in abundance of cells in the B cell compartment of WT or CD4-IRF4KO mouse strain (Figure 4A). This suggests that the observed changes in cytokine production in CD4-IRF4KO mouse during EAU cannot be attributed to B cells. On the other hand, intracellular cytokine staining analysis of cells in the eye or LN show significant expansion of CD4^+^ T cells producing IL-10 (Figure 4B) or IL-35 (Figure 4C). Surprisingly, we also detected a significant percentage of B cells expressing IL-35 in the spleen of CD4-IRF4KO compared to WT mice (Figure 4D). Collectively, these results indicate that loss of IRF4 expression in T cells altered overall pattern of cytokine secretion during EAU and that IRF4 produced by T cells might contributes to mechanisms that maintain T-helper cell homeostasis during sustained immunological response to pathogens.

### 2.5. Expression of IkZF and Th17 Signature Genes Is Suppressed in CD4-IRF4KO CD4^+^ T cells.

**Figure 5:**
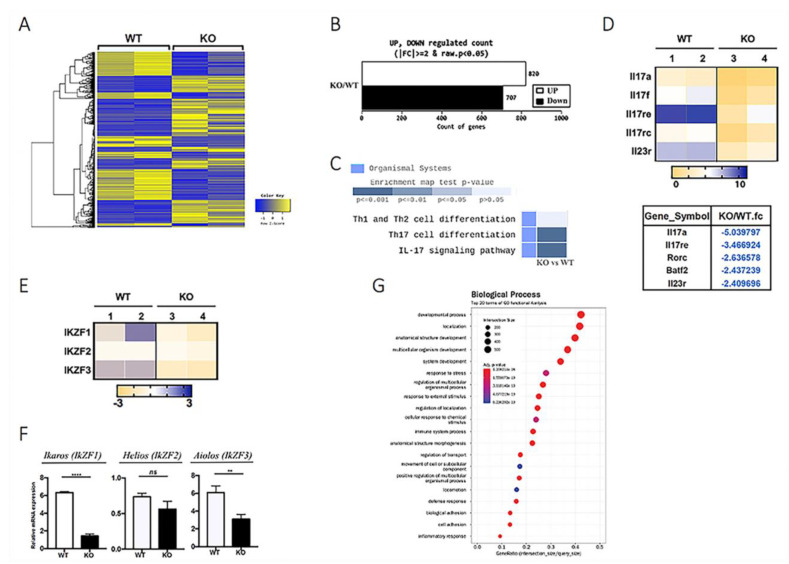
Mice lacking IRF4 in CD4^+^ T cells exhibit altered expression of Ikaros Zinc Finger (IKZF) and Th17 signature genes. RNA was isolated from control (WT) and CD4-IRF4KO CD4^+^ T cells at day 21 post-immunization with IRBP/CFA, and subjected to RNA-Seq analyses. (**A**,**B**) Heatmap derived from global RNA-Seq analysis identified upregulated and downregulated genes in IRF4-deficient T cells. (**C**) KEGG pathway analysis shows effects of IRF4 on the transcription of genes that regulate Th1, Th2 or Th17 differentiation. (**D**,**E**) Heatmaps reveal differential expression of Th17 signature (**D**) and IKZF (**E**) genes by WT and CD4-IRF4KO T cells. (**F**) RNA was isolated from control (WT) and CD4-IRF4KO T cells at day 21 post immunization with IRBP/CFA and subjected to qPCR analyses. (**G**) A Gene Ontology enrichment analysis of the differentially expressed genes was performed to evaluate enriched biological processes. Data represent at least three independent experiments. ** *p* < 0.01; **** *p* < 0.0001. ns: Not significant

IkZF family transcription factors and IRF4 have been shown to regulate T cell differentiation, and results from recent studies indicate that T cell expression of IRF4 during T cell development and lymphopoiesis is induced by IkZF factors [3,11,12,13,14,15]. We therefore immunized mice with IRBP_651–670_ in CFA to induce EAU, performed RNA-seq analysis using CD4^+^ T cells sorted at peak of disease and investigated the role of IRF4 in the regulation of transcription of genes required for T-helper cell differentiation, including genes that code for IkZF genes. Representative heatmap shows upregulation of 820 genes and downregulation of 707 genes in IRF4KO CD4^+^ compared to WT CD4^+^ T cells (Figure 5A,B). KEGG (Kyoto Encyclopedia of Genes and Genomes) pathway analysis revealed that while loss of IRF4 expression has less direct effects on genes that control Th1 or Th2 differentiation, it regulates transcription of genes that control Th17 differentiation program and signaling pathway (Figure 5C). Among the Th17 signature genes downregulated in CD4-IRF4 mice during EAU are *il17a, il17f, il17re, il17rc*, *rorc, batf2* and *il23r* (Figure 5D). It is notable that *ikzf1*, and to a lesser extent *ikzf3*, are downregulated in CD4-IRF4KO T cells, while transcription of other IkZF genes were not affected (Figure 5E,F). Furthermore, Gene Ontology (GO) analysis suggests that the gene expression pattern induced in absence of IRF4 had effects on several biological processes in the CD4-IRF4KO compared to WT counterpart (Figure 5G).

### 2.6. Cell Metabolism Is Altered in IRF4-Deficient T Cells

**Figure 6:**
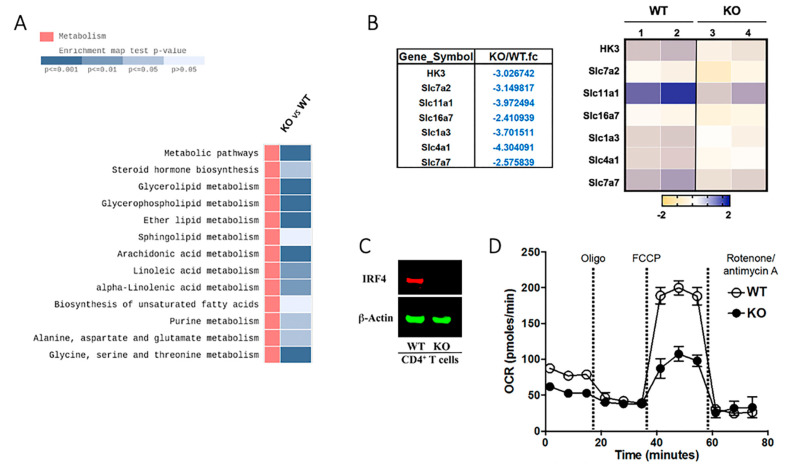
Loss of IRF4 in CD4^+^ T cells induced changes in cell metabolism during EAU. (**A**,**B**) CD4^+^ T cells derived from WT and CD4-IRF4KO mice during EAU were subjected to KEGG pathway analysis to determine whether loss of IRF4 expression in CD4^+^ T cells correlates with changes in the expression of metabolic gene. (**B**) Representative heatmap showing alterations in expression of metabolic pathway genes of WT and CD4-IRF4KO T cells. (**C**,**D**) Purified CD4^+^ T cells were isolated from WT or CD4-IRF4KO EAU mice (**C**) Images of the full-length Western blot gels are provided (Supplementary Figure S2). Glycolic assays were performed using the Seahorse Glycolytic Rate assay (**D**). Results are presented as time-dependent changes in oxygen consumption rate (OCR). Data represent three independent experiments.

The reduced numbers of T cells detected in the eyes of CD4-IRF4KO mice during EAU suggested alterations in bioenergetic mechanisms in the CD4-IRF4 T cells. We therefore sorted CD4^+^ T cells from WT or CD4-IRF4KO mice with EAU and examined whether the reduced proliferative capacity of CD4-IRF4KO T cells correlates with changes in glycolytic activity. CD4^+^ T cells sorted from the LN of WT or CD4-IRF4KO mice were also subjected to RNA-seq analysis. The representative heatmap shows dramatic changes in gene expression between the two mouse strains and KEGG pathway analysis revealed marked alteration in genes that regulate metabolic pathway in CD4-IRF4KO viz-a-viz WT T cells (Figure 6A). Of particular interest is the effects of IRF4-deficiency on the expression of genes that code for members of the solute carrier (SLC) transporter family. This large family of membrane transport proteins regulate diverse processes including cell metabolism. Compared to WT CD4^+^ T cells, the levels of several SLC family genes or the hexokinases-3 (HK3) gene that phosphorylates glucose are decreased in CD4-IRF4KO CD4^+^ T cells (Figure 6B). TCR activation enhances rapid generation of ATP which delivers glucose to the pentose phosphate pathway (PPP) for the generation of biosynthetic precursor molecules that sustain T cell proliferation. We sorted CD4^+^ T cells in the LN of IRBP-immunized mice WT or CD4-IRF4KO mice, established that the CD4-IRF4KO T cells were indeed deficient of IRF4 (Figure 6C) and then measure key parameters of mitochondrial function such as oxygen consumption rate (OCR) in the Agilent Seahorse XF system. The Cell Mito Stress Test detected significant decrease in basal mitochondrial respiration and maximal respiratory capacity in CD4-IRF4KO CD4^+^ T cells, suggesting decreased ability of the IRF4-deficient cells to optimally perform oxidative phosphorylation and ATP synthesis (Figure 6D). 

## 3. Discussion

Study of mutant mice with global deletion of *irf4* in all tissues (IRF4 knockout) revealed that IRF4 plays critical roles in regulating the development and functions of myeloid and B cells. However, the specific role of IRF4 in T lymphocytes is not well understood. In this study, we generated mice with targeted deletion of *irf4* in CD4^+^ T cell (CD4-IRF4KO) and investigated the specific functions of the IRF4 transcription in T cells. The IRF4-deficient T cells exhibit reduced capacity to induce expansion of T-helper subsets that cause autoimmune disease, suggesting that an intrinsic function of IRF4 in CD4^+^ T cells may be to sustain robust proinflammatory responses required to confer protection from pathogen. However, because CD4-Cre-mediated deletion of *irf4* occurs at the CD4^+^CD8^+^ double positive stage of T cell development in the thymus, effector CD8^+^ T cells of the CD4-IRF4KO mice do not express IRF4. Thus, biological effects observed in the CD4-IRF4KO mice cannot be attributed solely to CD4^+^ T cells but also CD8^+^ cytotoxic T cells. Importantly, as neither CD4^+^ nor CD8^+^ T cells express IRF4, we suggest that the CD4-IRF4KO mouse strain is ideally suited for investigating whether the expression of IRF4 is required for T cell mediated immune response. 

We therefore used the EAU model, an autoimmune disease confined to the immune privileged neuroretina, to investigate whether expression of IRF4 is necessary for the development of organ-specific autoimmune diseases mediated by T cells. Clinical evaluation of CD4-IRF4KO mice immunized with IRBP/CFA by Fundoscopy, histology and optical coherence tomography revealed development of hallmark features of uveitis characterized by massive infiltration of inflammatory cells into the retina and destruction of photoreceptor cells are significantly suppressed in the CD4-IRF4KO compared to WT mice. Dark- or light-adapted ERG recorded lower a- and b-wave amplitudes in the retina of WT mice consistent with visual impairment due to defects in cone and rod signaling functions. In contrast, the recorded a- and b-wave amplitudes were higher in CD4-IRF4KO and correlated with improved vision. Taken together, these clinical findings provide direct evidence that suppression of IRF expression in T cells can be exploited therapeutically. 

Amelioration of EAU in CD4-IRF4KO mice correlates with a significant decrease in percentage of cells expressing the Th17 lineage-specifying transcription factor, ROR-γt, particularly Th17 cells expressing IL-17 and DP-Th17 (IFN-γ^+^IL-17^+^) cells. The decrease in Th17 responses in IRBP-immunized CD4-IRF4KO mice is in line with previous reports that global deletion of *irf4* in mice dysregulates developmental program of several T-helper subsets, including Th17 cells [16,19]. On the other hand, increase of IFN-γ-producing Th1 cells in the eye and LN of the CD4-IRF4KO mice is also consistent with reports that IFN-γ contributes to the suppression of Th17 cells that mediate uveitis through activation of IL-27/STAT1 pathway [23]. Thus, the reduced level of Th17 with concomitant increase of Th1 cells in the eyes of CD4-IRF4KO mice during EAU underscores the requirement of IRF4 expression by T cells for pathogenesis of uveitis in mice. We also show that suppression of EAU pathology in CD4-IRF4KO correlates with expansion of IL-10-producing (Treg) or IL-35-producing (iTR35) T cells and production of these immune-suppressive cytokines play critical roles in downregulating immune responses that cause autoimmune diseases [23,27].

To provide a potential mechanistic explanation as to why the loss of IRF4 in T cells results in developmental defects in disparate T-helper subsets, we analyzed the transcriptomes of the WT or CD4-IRF4KO T cells. RNA-seq analysis of T cells isolated 21 days after induction of EAU revealed differential expression of genes that control lymphopoiesis and lymphocyte effector functions between the WT and CD4-IRF4 T cells. The RNA-seq data revealed that Ikaros Zinc Finger Transcription Factors (IkZF) that regulate T cell differentiation and metabolic programming are targets of IRF4. Ikaros and Aiolos were downregulated in CD4-IRF4KO T cells, while expression of Helios, Eos or Pegasus was not affected. This suggests that IRF4 is required for inducing Ikaros and Aiolos, the two IkZF members implicated in regulating TCR-mediated metabolic programming during inflammation [17]. Interestingly, the Cell Mito Stress Test showed decrease in basal mitochondrial respiration and maximal respiratory capacity in CD4-IRF4KO T cells, suggesting that the decreased ability to optimally perform oxidative phosphorylation and ATP synthesis may derived from deficit in Aiolos and Ikaros in CD4-IRF4KO T cells. These observations also imply that an IRF4/Ikaros axis might regulate T cell responses by controlling cell metabolism. Other clues to how expression of IRF4 might influence T-helper cell effector functions or lineage specification is informed by reports that TCR-induced signals activate and recruit pioneering transcription factors such as BATF and IRF4 to target genes such as *ikaros* or *aiolos* involved in regulating T-helper cell differentiation [28,29]. In context of the Th17 differentiation program, hypoxia stabilizes the Th17 lineage by promoting interaction between hypoxia-inducible factor-1 alpha (HIF1α) with ROR-γt while simultaneously destabilizing the Treg lineage through interaction of HIF1α with Foxp3 [29,30]. On the other hand, IRF4 is also thought to control ROR-γt-dependent Th17 inflammatory bowel disease by regulating *il17a* promoter activity [31]. Thus, the data presented here show that reduced EAU in CD4-IRF4KO mice is associated with decrease in Th17 cells and expansion of IL-10- or IL-35-producing regulatory T cells that produce, underscoring the role of IRF4 in regulating the levels of both effector and regulatory T-helper subsets during intraocular inflammatory diseases. 

Uveitis comprises a heterogeneous group of potentially sight-threatening inflammatory diseases that includes sympathetic ophthalmia, birdshot retinochoroidopathy, Behcet’s disease, Vogt-Koyanagi–Harada disease and ocular sarcoidosis. By some estimates, uveitis may account for more than 10% of severe visual handicaps in the United States [32,33]. The considerable impetus to develop new effective therapies for uveitis derives from the fact that conventional treatments including use of corticosteroids can cause serious side effects. The data presented in this study suggest that blockade of the IRF4 signaling pathway may be effective in suppressing expansion of Th17 cells and potentially blinding chronic uveitis characterized by remitting and recurring intraocular inflammation. 

## 4. Materials and Methods

### 4.1. Mice

We derived mice with conditional deletion of *Irf4* in CD4^+^ T cells (CD4-IRF4KO) by breeding *Irf4^fl/fl^* mice with CD4-Cre (Taconic, Hudson, NY, USA) mice. Littermate i*rf4^fl/fl^* mice on the C57BL/6J background, were used as wild type (WT) controls. Animal care and experimentation conformed to National Institutes of Health (NIH) guidelines. Mice were maintained and treated in accordance with National Eye Institute (NEI) and NIH Animal Care and Use Committee guidelines (Study # EY000262-19 & EY000372-14, 13 December 2019). The experimental protocol was approved under NIH/NEI Animal Study Protocol (ASP) # NEI-597.

### 4.2. Experimental Autoimmune Uveitis (EAU) 

EAU was induced by active immunization of C57BL/6J with IRBP_651-670_-peptide in a 0.2 mL emulsion (1:1 *v/v* with complete Freund’s adjuvant (CFA) containing *Mycobacterium tuberculosis* strain H37RA (2.5 mg/mL). Mice also received *Bordetella pertussis* toxin (1µg/mouse) concurrently with immunization [34]. Clinical disease was evaluated and scored by fundoscopy histology, optical coherence tomography (OCT) and electroretinography (ERG), as described previously [21,22,25]. 

### 4.3. RNA-seq

Total RNA was extracted using TRIZOL (Invitrogen, CA, USA) from CD4^+^ T cells from WT and CD4-IRF4KO mice after immunization. RNA integrity was checked by picogreen method using Victor X2 fluorometry. The extracted total RNA was processed to prepare the mRNA-sequencing library using the TruSeq stranded mRNA LT sample preparation kit according to TruSeq Stranded mRNA Sample Preparation Guide, Part #15031047 Rev. E. All samples were sequenced on a NovaSeq6000 S4 (150bp PE). The raw image data were transformed by base calling into sequence data and stored in FASTQ format. Trimmed reads are mapped to reference genome with HISAT2, splice-aware aligner and transcript is assembled by StringTie with aligned reads. Expression profiles are represented as read count and normalization value which is based on transcript length and depth of coverage. The FPKM (Fragments Per Kilobase of transcript per Million mapped reads) value or the RPKM (Reads Per Kilobase of transcript per Million mapped reads) value is used as a normalization value. Expression profiles are represented as read count and normalization value, which is based on transcript length and depth of coverage. The FPKM (Fragments Per Kilobase of transcript per Million mapped reads) value or the RPKM (Reads Per Kilobase of transcript per Million mapped reads) is used as a normalization value. All genes represent fold changes of at least 2.0 between the IRF4KO and WT groups. 

### 4.4. Western Blot Analysis

Cells were lysed in RIPA buffer (10 mM Tris-Cl (pH 8.0), 1mM EDTA, 1% of Triton X-100, 0.1% sodium deoxycholate, 0.1% SDS, 140 mM NaCl and 1mM PMSF) and lysates were incubated for 30 min at 4 °C. After incubation, lysates were centrifuged at 14,000× *g* rpm for 30 min and supernatants were harvested. Lysates (10 μg/lane) were fractionated on 4–12% gradient SDS-PAGE, and the antibodies used were: IRF4 (cell signaling, Danvers, MA, USA) and β-actin (cell signaling). After secondary antibody reaction, signals were detected with LI-COR system (LI-COR Biosciences, Lincoln, NE). Image studio software (LI-COR Biosciences, Lincoln, NE) was used for data analysis. 

### 4.5. Fundoscopy 

Funduscopic examinations were performed at day 21 after EAU induction. Fundus image was captured using Micron III retinal imaging microscope (Phoenix Research Labs, Pleasanton, CA, USA) for small rodent or a modified Karl Storz veterinary otoendoscope coupled with a Nikon D90 digital camera, as previously described [22,35]. The need to avoid a subjective bias was obviated by evaluating fundus photographs without knowledge of mouse identity and by masked observers. At least six images (two posterior central retinal view, four peripheral retinal views) were taken from each eye by positioning the endoscope and viewing from superior, inferior, lateral or medial fields and each lesion was identified, mapped and recorded. Clinical grading of retinal inflammation was performed as established [21,36,37].

### 4.6. Histology

Eyes for histology were enucleated, fixed in 10% buffered formalin and serially sectioned in the vertical pupillary-optic nerve plane. Specimens were then dehydrated through graded alcohol series, embedded in methacrylate, serial transverse sections (4 µm) cut and stained with hematoxylin and eosin (H&E). Photographs of representative sections are taken on a photomicroscope.

### 4.7. Optical Coherence Tomography (OCT) 

Optical coherence tomography (OCT) is a noninvasive procedure that allows visualization of internal microstructure of various eye structures in living animals. Mice were then immobilized using an adjustable holder that allowed for horizontal or vertical scan scanning, and each scan was performed at least twice, with realignment each time. The dimension of the scan (in depth and transverse extent) was adjusted until the optimal signal intensity and contrast were achieved. Retinal thickness was measured from the central retinal area of all images obtained from both horizontal and vertical scans from the same eye, using the system software, and averaged. The method used to determine the retinal thicknesses in the system software was as described in [34,38].

### 4.8. Electroretinogram (ERG) 

ERG measures change in electrical potentials in response to light stimulation of the retina and is used to identify gross physiologic changes, which are pathognomonic for visual function defects. Before ERG recordings, mice were dark-adapted overnight, and experiments were performed under dim red illumination. ERG was recorded on anesthetized mice using an electroretinography console that generates and controls the light stimulus. Dark-adapted ERG was recorded with a single flash delivered in a Ganzfeld dome, with a reference electrode (gold wire) placed in the mouth and a ground electrode (subcutaneous stainless-steel needle) positioned at the base of the tail. Signals were differentially amplified and digitized. Amplitudes of the major ERG components (a- and b-wave) were measured using automated methods [21].

### 4.9. Metabolic Analysis

Isolated CD4^+^ T cells (0.5 × 10^6^ cells) from WT- and IRF4KO-immunized mice were seeded onto a Seahorse XFp cell culture Miniplate and then incubated for 16 h. After incubation, cells were washed with XF assay medium and incubated in non-CO_2_ incubator for 1 h. In addition, we measured Oxygen consumption rate (OCR) using Seahorse Extracellular Flux analyzer (Agilent Technologies, Santa Clara, CA, USA) according to the manufacturer’s kit protocol (Cell Mito Stress Test Kit, Agilent Technologies).

### 4.10. Flow Cytometery

For intracellular cytokine detection, cells were restimulated for 4  h with PMA (20  ng/mL)/ionomycin (1  µM). GolgiStop was added in the last hour, and intracellular cytokine staining was performed using BD Biosciences Cytofix/Cytoperm kit as recommended (BD Pharmingen, San Diego, CA, USA). FACS analysis was performed on a CytoFLEX Flow Cytometer (Beckman Coulter, Indianapolis, IN, USA) using protein-specific monoclonal antibodies and corresponding isotype control Abs (BD Pharmingen, San Diego, CA, USA), as described previously [39]. FACS analysis was performed on samples stained with mAbs conjugated with fluorescent dyes, and each experiment was color compensated. Dead cells were stained with dead cell exclusion dye (Fixable Viability Dye eFluor^®^ 450; eBioscience, San Diego, CA, USA), and live cells were subjected to side scatter and forward scatter analysis. Quadrant gates were set using isotype controls with less than 0.2% background.

### 4.11. Statistical Analysis

Data analysis was performed using non-parametric methodology that are not based on normal distribution of the data. We also used Students’s *t*-test for analysis of data as mean ± SEM.

## Data Availability

All datasets generated for this study are included in the article/supplementary material.

## Supplementary Materials

The following are available online at https://www.mdpi.com/1422-0067/22/5/2775/s1.
