# Supplementary material for: Deletion of Irf4 in T Cells Suppressed Autoimmune Uveitis and Dysregulated Transcriptional Programs Linked to CD4+ T Cell Differentiation and Metabolism
Source: Int J Mol Sci. 2021 Mar 9;22(5):2775. doi: 10.3390/ijms22052775 (PMC7967141; doi:10.3390/ijms22052775)
Supplement: Supplementary file 1 [file ijms-22-02775-s001.pdf]

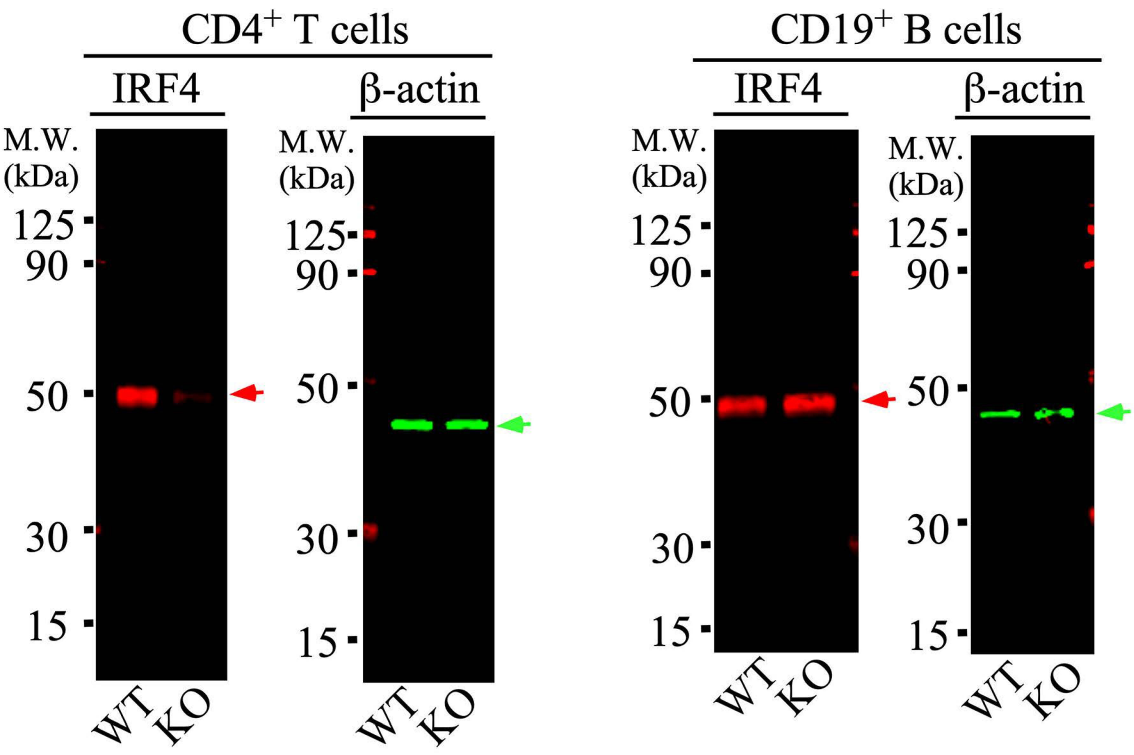

Supplementary Figure S1. Characterization of mice with targeted deletion of irf4 in T cell. $Irf4^{fl/fl}$ mice by Western blot analysis. Figure represents full-length gels of image presented as Fig. lB.

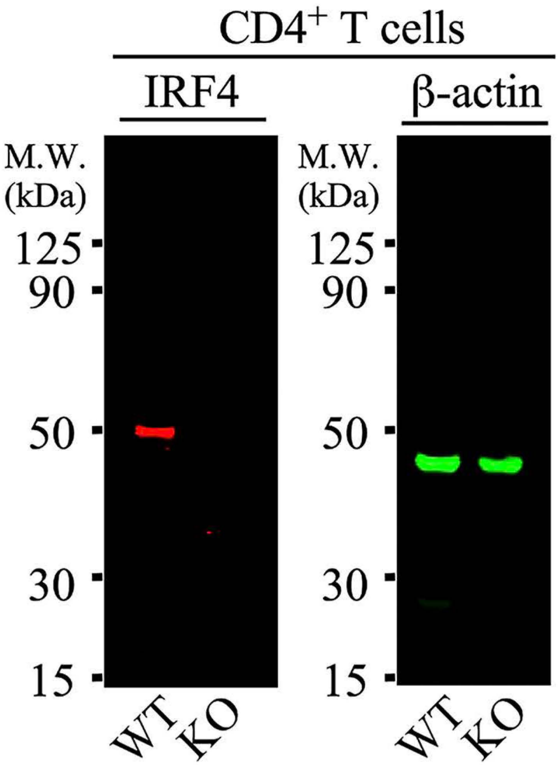

Supplementary Figure S2. Characterization of mice with targeted deletion of irf4 in T cell. Irf4$^{fl/fl}$ mice by Western blot analysis. Figure represents full-length gels of image presented as Fig. 6C.
